# Impact of early psychosocial factors (childhood socioeconomic factors and adversities) on future risk of type 2 diabetes, metabolic disturbances and obesity: a systematic review

**DOI:** 10.1186/1471-2458-10-525

**Published:** 2010-09-01

**Authors:** Teresa Tamayo, Herder Christian, Wolfgang Rathmann

**Affiliations:** 1 Institute of Biometrics and Epidemiology, German Diabetes Center, Leibniz Center for Diabetes Research at Heinrich-Heine-University, Düsseldorf, Germany; 2 Institute for Clinical Diabetology, German Diabetes Center, Leibniz Center for Diabetes Research at Heinrich-Heine-University, Düsseldorf, Germany

## Abstract

**Background:**

Psychological factors and socioeconomic status (SES) have a notable impact on health disparities, including type 2 diabetes risk. However, the link between childhood psychosocial factors, such as childhood adversities or parental SES, and metabolic disturbances is less well established. In addition, the lifetime perspective including adult socioeconomic factors remains of further interest.

We carried out a systematic review with the main question if there is evidence in population- or community-based studies that childhood adversities (like neglect, traumata and deprivation) have considerable impact on type 2 diabetes incidence and other metabolic disturbances. Also, parental SES was included in the search as risk factor for both, diabetes and adverse childhood experiences. Finally, we assumed that obesity might be a mediator for the association of childhood adversities with diabetes incidence. Therefore, we carried out a second review on obesity, applying a similar search strategy.

**Methods:**

Two systematic reviews were carried out. Longitudinal, population- or community-based studies were included if they contained data on psychosocial factors in childhood and either diabetes incidence or obesity risk.

**Results:**

We included ten studies comprising a total of 200,381 individuals. Eight out of ten studies indicated that low parental status was associated with type 2 diabetes incidence or the development of metabolic abnormalities. Adjustment for adult SES and obesity tended to attenuate the childhood SES-attributable risk but the association remained. For obesity, eleven studies were included with a total sample size of 70,420 participants. Four out of eleven studies observed an independent association of low childhood SES on the risk for overweight and obesity later in life.

**Conclusions:**

Taken together, there is evidence that childhood SES is associated with type 2 diabetes and obesity in later life. The database on the role of psychological factors such as traumata and childhood adversities for the future risk of type 2 diabetes or obesity is too small to draw conclusions. Thus, more population-based longitudinal studies and international standards to assess psychosocial factors are needed to clarify the mechanisms leading to the observed health disparities.

## Background

In adults, adverse psychosocial factors such as low socioeconomic status (SES), deprivation and traumata have been shown to be associated with type 2 diabetes [1], obesity [2-5], cardiovascular disease [6,7], and unhealthy lifestyle habits [8-11]. A lower social status is related to higher stress levels [12] and poor living conditions [13], which may partly explain these associations. Health disparities can be observed fairly early with lower birth weights, an earlier adiposity rebound and higher rates of infant mortality in the low SES groups [14-16].

Data on the association of type 2 diabetes and adverse childhood circumstances is more limited [17]. In two cross-sectional analyses there was evidence that adverse social conditions in childhood are independently associated with an increased risk of metabolic impairments and insulin resistance [18,19]. In a Swedish population-based study, Agardh et al. found low parental education, low family household income and low parental occupational position to be associated with a more than two-fold increased diabetes risk in adulthood, which was attenuated after accounting for adult socioeconomic factors [20].

Thus, the primary aim of this review was to evaluate the risk of psychosocial factors on type 2 diabetes incidence and the role of change in socioeconomic conditions throughout life on the basis of population-based and longitudinal studies. As several factors of childhood environment are closely related and interact with each other [21], we aimed not only to address the relationship of childhood socioeconomic status (CSES) with diabetes incidence, but to investigate a broad range of psychosocial factors. Furthermore, obesity was included as it is considered a key factor for diabetes incidence in youth and as it is also influenced by SES [22]. Since a first comprehensive review [23] on the inverse relationship between SES and obesity in developed countries, these early results have been substantiated in further systematic reviews [2,4]. We included obesity as endpoint in our review and concentrated on longitudinal and population-based studies with particular focus on the analysis of change in weight status and psychosocial circumstances.

## Methods

### Search criteria

We searched Medline in July 2008 and carried out an update of the search in April 2010 applying the search algorithm "(diabetes or insulin resistance or prediabetes or metabolic syndrome) and (SES or social or socioeconomic or psychosocial or income or working status or migration or community or adversities or deprivation or depression or abuse or high risk family or hostility)".

For obesity a search in Medline was carried out in November 2008 (updated in April 2010) using the algorithm" (obesity or overweight) and (socioeconomic or social or deprivation or adversities or childhood socioeconomic or family environment or early life or youth or childhood adversities or deprivation) and (longitudinal or prospective or cohort)".

Both searches were limited to publications after 31 December 1994 in English language. During the 1990 s, the diabetes criteria were changed several times, which complicates comparison with earlier publications.

### Inclusion and exclusion criteria

Studies were included if incident cases of type 2 diabetes were assessed. Cross-sectional studies with data on diabetes prevalence only were excluded. Only population- or community-based studies were included to have a more homogeneous database for this review. Further outcome criteria were insulin resistance, elevated HbA1c values, and the metabolic syndrome because they share risk factors and pathophysiological pathways with type 2 diabetes. Obesity and overweight are considered key risk factors for type 2 diabetes incidence especially in youth, and were furthermore included as outcome parameters, but were analysed separately.

### Risk factor specification: Childhood psychosocial factors

Studies on incident type 2 diabetes or obesity were included if they contained data on psychosocial factors including a wide range of indicators reflecting the social and psychological conditions under which the participants grew up:

(1) basic indicators of CSES: parental education; parental occupation and family income as indicators of economic wealth and stability;

(2) further indicators of wealth and deprivation: public housing, housing conditions, house ownership, unemployment;

(3) the high-risk family concept (adverse childhood experiences) regarding neglect, abuse and household dysfunction [24];

(4) indicators of impaired psychological health in children such as depression or anxiety;

(5) indicators of the ability to cope with stressful conditions like coping skills or sense of control [25];

(6) indicators of possibly stressful situations: migration, parental stress (e.g. stressful working conditions or parenting stress which are supposed to have implications for children's stress as well);

(7) neighbourhood deprivation indexes as indicator for the aggregation of unfavourable circumstances clustered in residential areas. These indexes are based on the social composition of neighbourhoods regarding for example the social status of its habitants, housing and street conditions, mean household sizes per person and other indicators of wealth such as mean household number of cars [26,27].

Studies were excluded if information on obesity and overweight was only available at one time point and no adjustment for previous weight status was done. Studies, which only included type 1 diabetes were also excluded. Self-reported diabetes type may not offer a reliable distinction between the diabetes mellitus types. However, self-reported diabetes type was found in most studies and as type 1 diabetes contributes to a fairly small proportion of the total number of diabetes cases we accepted self-reported diabetes type for the assessment of type 2 diabetes incidence.

### Data extraction and quality management

Eligible studies were assessed by one reviewer (T. T.) and discussed with a second reviewer (W. R.). The final list of variables extracted from the selected studies contained the first author, publication year, country where the study was carried out, cohort size, study duration, age characteristics of participants, measurement of risk factors, definition of diabetes/obesity variables and a brief description of results including effect size. For missing information we contacted some of the authors of the selected studies. Any disagreements regarding numbers, study inclusion, and further analysis were resolved by consensus between the authors (T.T., C.H., W.R.).

### Analysis and quality assessment

We restricted the analysis to descriptive measures because of the lack of statistical comparability for most studies. Effect sizes extracted from the publications reflect adjusted results for odds ratio (OR), hazard ratio (HR) and relative risk (RR) regarding confounding (1) for age, sex, BMI, physical activity, smoking and alcohol (for type 2 diabetes incidence) or (2) for birth weight and adult SES (for obesity and overweight). We developed a 5 item quality scale adapted to our purpose with reference to methodological recommendations of the Meta-analysis Of Observational Studies in Epidemiology (MOOSE) group [28,29]. The sum score derived from this scale mainly had to reflect the study design, the examined outcome parameters, the risk factor construction and the control for confounders. Quality was judged taking into account the current state of research on type 2 diabetes and obesity and possible sources of bias. The following criteria were included into the quality score for the (1) diabetes and (2) obesity studies:

• (1&2) Study Duration (D): Highest quality was considered to be obtained from birth cohorts (D = 1) as the future diabetes and obesity risk is influenced strongly by very early health indicators such as birth weight and gestational age. Furthermore, especially in childhood body composition and metabolic functioning vary in different age groups, so that homogeneous age groups as accomplished by birth cohort studies offer another quality advantage [30,31].

• (1&2) Recruitment (R): Our inclusion criteria comprised only studies from which a high grade of representativeness and reproducibility was expected. To further assess the quality of recruitment in the score, population-based or school-based studies with characteristics of a census were rated as of highest quality (R = 1).

• (1&2) Explanatory variables/risk factor specification (E): We expected a source of bias from retrospectively assessed childhood psychosocial factors in the offspring (E = 0). In case parents gave direct information on their occupational, educational and financial situation we rated the quality of risk factor construction as high (E = 1).

• (1) Outcome parameter diabetes: Highest evidence was expected from blood glucose measurements with internationally valid cut off-levels as defined by the American Diabetes Association or WHO (O = 1) [32]. Self-reported diabetes leads to an underestimation of type 2 diabetes cases due to a high number of undiagnosed cases [33,34]. Therefore, we considered the quality based on self-report as low (O = 0). The plasma-based measure of insulin resistance (HOMA-IR calculated from fasting glucose and insulin levels) is closely related to (pre)diabetes. Thus, evidence from HOMA-IR-based data were considered as high (O = 1). Although HbA1c has been suggested as diagnostic tool for type 2 diabetes (ADA 2010), especially in early diabetes and in prediabetes, HbA1c values lead to misinterpretation which may be also due to a genetic component in the metabolisation of glycated haemoglobin [35-39]. Thus, we rated the quality of HbA1c values as low (O = 0). Metabolic syndrome and type 2 diabetes share some risk factors, but are too different as entities to include the metabolic syndrome as surrogate for type 2 diabetes. We allowed for the term 'metabolic' during Medline search, but rated metabolic syndrome as outcome criteria of low evidence for our study question (O = 0).

• (2) Outcome parameter obesity (O): First, measured weight and height to calculate BMI met our quality demands. Self-reported weight and height on the other hand have been reported to be imprecise in adults and in parents reporting anthropometric data of their children. Standard cut-off values for overweight and obesity served as second indicator for high quality: In children overweight and obesity are defined based on the age and sex-specific 85^th ^and 95^th ^BMI percentiles in growth charts from the National Health and Nutrition Examination Survey (NHANES), the Centers for Disease Control and Prevention (CDC) or the International Obesity Taskforce (IOTF), whereas in adults cut-off values for BMI ≥ 25 kg/m² for overweight and ≥ 30 kg/m² for obesity were used. The highest score (O = 1) was given only if both criteria were fulfilled.

• (1&2) Confounding (C): Quality of adjustment for confounders was inferred from important behavioural pathways leading to type 2 diabetes. Adjustment of age, sex, BMI, smoking, physical activity and alcohol consumption was required for the highest quality score (C = 1). Adjustment for obesity in the extracted studies was heterogeneous and depended highly on the study question. According to the basic requirements for our review question a high study quality (O = 1) demanded at least control for birth weight or baseline BMI to have possible weight change considered and the adjustment for adult SES to control SES change throughout life.

## Results

### Identification of relevant studies

**Figure 1:**
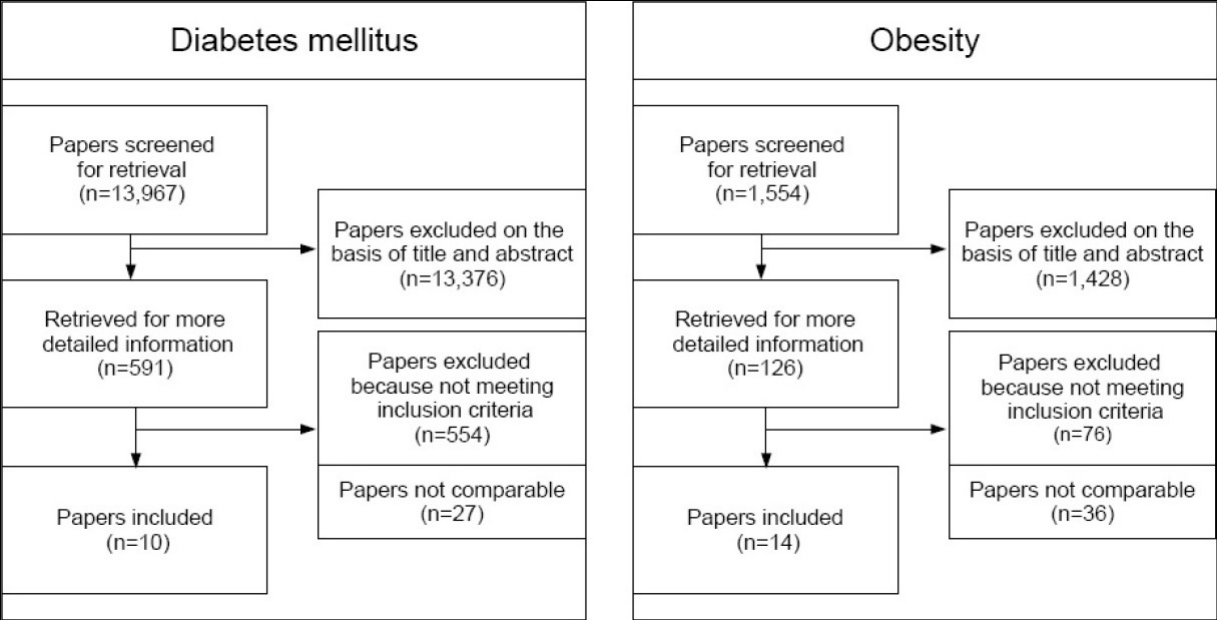
**Flow diagram of systematic review on type 2 diabetes incidence and on obesity**.

The search strategy yielded a total of 19,504 results. After exclusion of 5,537 non-English articles, reviews, animal studies, case reports and articles unrelated to type 2 diabetes, 13,967 abstracts were screened (see also Figure 1). We assumed that social parameters are often treated as confounders and in this case the results are presented in full text articles, but not in abstracts. Therefore, the first screening of abstracts served mainly to identify articles on diabetes incidence (591). Of these, 280 did not meet the study design criteria and were excluded. The further thematic evaluation of the psychosocial relevance of the studies was based on full text articles. Of these 273 were thematically irrelevant mainly because they did not offer information on childhood psychosocial factors. One study retrieved during the update of the search was not added, because it presented race stratified results and there was already non-stratified data available from this study from the first search [40].

For reasons of limited statistical comparability (p.eg. limited presentation of data) 10 studies remained for further descriptive analysis (Table 1). Information on excluded studies with a unique method of analysis such as path models is provided in the discussion, information on excluded studies with solely examined risk factors in the results.

**Table 1:** Overview of studies on childhood psychosocial factors and incident type 2 diabetes

**No**.	Authors	Year	Nation/ **Abbr**.	N	Age at baseline	Follow up (years)	Risk factor	Outcome	Effect size (95% CI or SE) (adjusted)		Study quality score^1^
D1 [41]	Lidfeldt et al.	2007	USA NHS	100,330 females	30-55	22	Father´s occupation 5 levels^a^	**T2DM **(FPG > 140 mg/dl)˚	RR (CI) laborer vs. rest ^a^	1.08 (0.95; 1.2)	1 (D = 0; R = 0; E = 0; O = 0, C = 1)
D2 [42]	Maty et al.	2008	USA ACS	5,913	17-94	34	Father´s occupation 2 levels manual/non-manual	Self-reported **DM**	HR (CI) for manual vs. non-manual	m 1.2 (0.8; 1.7) w 1.7 (1.2; 2.4)	3 (D = 1; R = 1; E = 0; O = 0; C = 1)
D3 [43]	Gissler et al.	1999	FIN	59,865	0	7	Mother´s occupation 3 levels^b^	**DM **criteria register based	OR (CI) for blue-collar vs. upper white collar^b^	0.83 (0.5; 1.5)	3 (D = 1; R = 1; E = 1; O = 0; C = 0)
D4 [49]	Hayes et al.	2006	UK	233 (BMI≥25)	0	50	Father´s occupation 3 levels^c^	**"metabolically normal" **(MetS)	OR (CI) for Class IV, V vs. I, II	m 0.2 (0.05; 0.8)* w 0.6 (0.1; 2.7)	1 (D = 1; R = 0; E = 0; O = 0; C = 0)
D5 [47]	Langen-berg et al.	2006	UK	2,629	0	53	Father´s occupation 6 levels^d^	**HbA1c **( > 5,8%)	OR (CI) for lowest vs. highest class	m 1.1 (1.0; 1.8) w 0.8 (0.5; 1.4)	3 (D = 1; R = 1; E = 1; O = 0; C = 0)
D6 [46]	Kivimäki et al.	2005	FIN	1,922	3-18	21	Parental occupation 3 levels^b ^	**HOMA-IR**	OR (CI) for change per descending CSES category	m 1.3 (1.03; 1.6)* w 1.2 (0.98; 1.5)	4 (D = 1; R = 1; E = 1; O = 1; C = 0)
											

D7 [44]	Kohler et al.	2005	MEX	6,423	50+	2	Parental education 4 levels^e^	Self-reported **DM**	OR (CI)^h ^for mother > elementary vs. rest	0.6 (0.5; 0.8)**	2 (D = 0; R = 1; E = 1; O = 0; C = 0)
D8 [45]	Best et al.	2005	USA HRS	12,589	51+	4	Parental education (in years)	Self-reported **DM**	β (SE) regression coefficients (linear)	m 0.2 (0.1) w 0.05 (0.1)	2 (D = 0; R = 1; E = 1; O = 0; C = 0)
D9 [50]	Goodman et al.	2007	USA PSD	1,167	13-19	3	Parental education; 4 categories^f^	**HOMA-IR**	β (SE) for high school or less vs. rest	4.5 (0.78)**	3 (D = 1; R = 0; E = 1; O = 1, C = 0)
											

D10 [48]	Thomas et al.	2008	UK	9,310	0	45	Childhoood adversity**^g^**	**HbA1c **( > 6,0%); **MetS**	OR (CI) for mother: little interest in education	1.4 (0.99; 1.9)	3 (D = 1; R = 1; E = 0; O = 0; C = 1)

NHS: Nurse's Health Study

ACS: Alameda County Study

HRS: Health and Retirement Study

PSD: Princeton School District Study

T2DM: Type 2 Diabetes mellitus

DM: Diabetes mellitus

MetS: Metabolic Syndrome

HbA1c: Glycated Hemoglobin

HOMA-IR: Homeostatic Model Assessment - Insulin Resistance

Β: Beta Coefficient

HR: Hazard Ratio

OR: Odds Ratio

RR: Relative Risk

CI: Confidence Interval

SE: Standard Error

^1 ^D Duration (1 = birth cohort or starting in youth)

R Recruitment (1 = population-based or school-based)

E Explanatory variables, (1 = risk factor assessment for CSES directly in parents, not retrospectively in adults)

O Outcome type 2 diabetes (1 = according to ADA diagnostic criteria for fasting plasma glucose)

C Confounding (1 = adjustment of age, sex, BMI, physical activity, smoking and alcohol)

^a ^Edwards classification of social class: upper white-collar, lower white-collar, blue-collar, laborers, farmers

^b ^As classified by Statistics Finland, 1989: upper non-manual, lower non-manual, manual,

^c ^UK Regristrar General's Standard Occupational Classification: I u. II most advantaged; III manual; IV u. V least advantaged.

**^d ^**Professional, intermediate, skilled nonmanual, skilled manual, semiskilled, unskilled.

**^e ^**Without any; some elementary; completed elementary; more than elementary education (≥7years)

**^f ^**Without any formal education, high school or less, some college, college graduate or higher.

^g ^Questionnaire including abuse, physical & emotional neglect, household dysfunction

^h ^Confidence interval self-calculated from standard error.

m = men; w = women

˚ ADA diagnostic criteria for type 2 diabetes mellitus: fasting plasma glucose (FPG) ≥ 126 mg/dl

** p < 0.01/* p < 0.05

The second search strategy for the outcome obesity retrieved 1,631 results (see also Figure 1). 77 reviews and non-English publications were excluded. 1,428 studies were classified as thematically irrelevant and 76 publications did not meet study design criteria. This led to 50 longitudinal studies of which 36 were not comparable for methodological reasons and risk factor selection. Hence, 14 publications (13 studies) were included for this review and are presented in Table 2.

**Table 2:** Overview of studies on childhood psychosocial factors and overweight and obesity

No.	Authors	Year	Nation/Abbr.	N	Age at baseline	Follow up (years)	Risk factor	Outcome	Effect size (95%CI or SE) (adjusted)		**Study quality score**^**1**^
O1 [67]	Giskes et al.	2008	NL GLOBE	1,465	40-60	13	Father´s occupation blue-, white collar, professional	**Self-reported **weight, height², BMI change	OR (CI) blue vs. professional	m 0.9 (0.6; 1.4) w 2.8 (1.6; 5.2)	1 (D = 0; R = 1; E = 0; O = 0; C = 0)
O2 [65]	Kristensen et al.	2006	DK EHYS	384	8-10 (3^rd ^grade)	6	Mother´s occupation blue-white collar	**Measured **weight, height³, Developing overweight at T2	OR (CI) blue vs. white collar	2.4 (1.02; 5.4)	4 (D = 1; R = 1; E = 1; O = 1; C = 0)
O3 [54]	Novak et al.	2005	SE	1,044	16	14	Father´s occupation blue-white collar	**Measured **weight, height (16y)^4^/self-reported (30y)²	OR (CI) blue vs. white collar	m 1.4 (0.7; 2.6) w 1.4 (0.8; 1.4)	4 (D = 1; R = 1; E = 1; O = 0; C = 1)
O4 [66]	Elgar et al.	2005	UK HBSC	355	12.3 (mean)	4	Parental occupation^b^	**Measured **weight, height^4^	β (SE)	-0.05 (0.08)	4 (D = 1; R = 1; E = 1; O = 0; C = 1)
O5 [55]	Power et al.	2003	UK	11,405	0	33	Father´s occupation 5 levels ^a^	**Self-reported **weight, height; 90^th ^intracohort percentile	OR (CI), continuous variable	m 1.04 (0.9; 1.2) w 1.10 (1.07; 1.1)	3 (D = 1; R = 1; E = 1; O = 0; C = 0)
O6 [56]	Laitinen et al.	2001	FIN	6,279	0	31	Father´s occupation 5 levels ^b^	**Measured **height, weight at 14y^6^, at 31y^2^	β (SE) social class VI vs. III^b^	m 0.05 (0.16) w 0.48 (0.17)	4 (D = 1; R = 1; E = 1; O = 0; C = 1)
								Self-reported weight and height; IOTF cut-points for obesity in youth; BMI ≥30 kg/m² in adulthood Difference in **age at onset of obesity**			3 (D = 1; R = 1; E = 0; O = 0; C = 1)

O7 [68]	Lee et al.	2009	USA Add Health	9,730	12-19	7	Parental education 2 levels </≥high school	**measured **weight, height;^2,3 ^become obese, stay obes	β (SE) stay obese	m 0.467(0.147)** w 0.481(0.152)**	4 (D = 1; R = 1; E = 1; O = 1; C = 0)
O8 [60]	Koupil et al.	2007	SE	6,535 (male conscripts)	18.2 (mean)	18	Maternal education 3 levels;^c ^(register data)	Measured height, weight at 18y.²	OR (CI) lowest vs. highest	1.03 (0.6; 1.8)	4 (D = 1; R = 0; E = 1; O = 1; C = 1)
O9 [57]	Salsberry et al.	2007	USA NLSCM	3,368	0	13	Maternal education metric- number of years.	Measured height, weight.^5,7 ^	OR (CI)	0.97 (0.9; 1.03)	5 (D = 1; R = 1; E = 1; O = 1; C = 1)
O10 [58]	Dubois et al.	2006	CDN QLSCD	1,550	0	4,5	Maternal education 4 levels^d^	Measured weight, height.^5^	OR (CI) lowest vs. highest	1.4 (0.8; 2.4)	5 (D = 1; R = 1; E = 1; O = 1; C = 1)
O11 [61]	Strauss et al.	1999	USA NLSY	2,913	0-8	6	Maternal education 3 levels^f^	Measured height, weight; obesity incidence^6^	RR (CI) lowest vs. highest	0.96 (0.7; 1.4)	4 (D = 1; R = 1; E = 0; O = 1; C = 1)
[69]	Ballistreri et al.	2009	USA ECLS-K	12,696	5-6 (Kindergarten)	6	Parental education 3 levels^g^	Measured height, weight, BMI growth curve models	β (SE) for growth in BMI	0.007* (0.003)	4 (D = 1; R = 1; E = 1; O = 1; C = 0)
[59]	Howe et al.	2010	UK ALSPAC	7,772	0	15-16	Maternal education 4 levels^h^	DXA- assessed total fat mass	SII (CI)	m 1.21 (1.08;1.36) w 1.34 (1.23;1.46)	4 (D = 1; R = 1; E = 1; O = 1; C = 0)
								Self-reported weight and height; IOTF cut-points for obesity in youth; BMI > = 30 in adulthood Difference in **age at onset of obesity**			3 (D = 1; R = 1; E = 0; O = 0; C = 1)

O12 [62]	Gordon-Larson et al.	2007	USA Add Health	14,654	12-19	7	Family income </≥ 26.000 US$	Self-reported weight and height;^2,3 ^age at onset of obesity	β (CI) onset low vs. high income	-2.6 (-3.8; -1.3)**	3 (D = 1; R = 1; E = 1; O = 0; C = 0)
O13 [57]	Salsberry et al.	2007	USA NLSCM	3,368	0	13	Family income lifetime and 1990 per capita income in US$	Measured height and weight;^5,7^	OR (CI)	1.0 (0.99; 1.00)	5 (D = 1; R = 1; E = 1; O = 1; C = 1)
O14 [58]	Dubois et al.	2006	CDN QLSCD	1,550	0	4,5	Family income </≥20000 CN$/y	Measured weight, height; Obesity ^5^	OR (CI) < vs. ≥ 20000 CN$	2.5 (1.3; 4.8)**	5 (D = 1; R = 1; E = 1; O = 1; C = 1)
O15 [61]	Strauss et al.	1999	USA NLSY	2,913	0-8	6	Family income intracohortal 15^th^/85^th ^percentile	Measured height, weight; obesity incidence^6^	RR (CI) lowest vs. highest family income	2.8 (1.4; 5.8)	4 (D = 1; R = 1; E = 0; O = 1; C = 1)
O16 [69]	Ballistreri et al.	2009	USA ECLS-K	12,696	5-6 (Kindergarten)	6	Log transformed yearly Family income in US$	Measured height, weight, BMI growth curve models	β (SE) for growth in BMI	0.003 (0.001)	4 (D = 1; R = 1; E = 1; O = 1; C = 0)

GLOBE: Gezondheid en LevensOmstandigheden Bevolking Eindhoven en omstreken (health and living conditions of the population of Eindhoven and its surroundings)

EHYS: Danish part of the European Youth Heart Study

HBSC: Health Behaviour in School-aged Children

NLSY: The National Longitudinal Surveys

ECLS-K: Early Childhood Longitudinal Study, Kindergarten Class of 1998-1999

ALSPAC: Avon Longitudinal Study of Parents and Children

NLSCM: National Longitudinal Surveys Child-Mother files

QLSCD: Longitudinal Study of Child Development in Québec

SII: Slope Index of Inequalities (linear regression coefficient and CI presented) [93]

Β: Beta Coefficient

HR: Hazard Ratio

OR: Odds Ratio

RR: Relative Risk

CI: Confidence Interval

SE: Standard Error

^1 ^D Duration (1 = birth cohort or starting in youth)

R Recruitment (1 = population-based or school-based representing regional population at certain age)

E Explanatory variables, (1 = risk factor assessment for CSES directly in parents, not retrospectively in adults)

O Outcome (1 = measured BMI and age-specific cut off scores for obesity/overweight criteria (extra-cohortal definition))

C Controlling (1 = adult SES, birth weight or baseline BMI)

²overweight/obesity criteria for adults: BMI ≥ 25 kg/m²/≥ 30 kg/m²;

³ overweight/obesity criteria: IOTF age specific BMI cut points

^4^overweight BMI cut points according to Cole (BMJ, 2000)

^5 ^age-specific 85/95^th ^percentile (CDC growth charts)

^6 ^age-specific 85/95^th ^percentile, (NHANES)

^7 ^partly self-reported height, weight with controlling for method of data collection

^a ^I+II skilled nonmanual; III unskilled nonmanual; skilled manual IV+V semi- unskilled manual

^b ^I+II professional; III skilled workers IV unskilled workers; V farmers. UK Regristrar General's Standard Occupational Classification

^c ^elementary; secondary; post-secondary

**^d ^**No high-school; high school; college; university degree

**^e ^**No high-school; high school; some college; college graduate, professional degree

**^f ^**less than high school; high school only; college or professional

**^g ^**less than high-school; some college; college

**^h ^**< O-level, O-level, A-level, >A-level

m = men; w = women

** p < 0.01/* p < 0.05

### Description of included studies: diabetes incidence

#### Study design

Five publications involving 9,200 cases of incident diabetes were included [41-45]. Furthermore, we identified 147 cases of insulin resistance [46], 953 cases of elevated HbA1c [47,48] and 233 cases of "metabolic malfunctioning" [49] in a total of 199,214 individuals. One study analysed HOMA-IR as continuous variable in 1,167 individuals, but did not present the number of cases with insulin resistance [50]. Hence, overall 10 studies are summarised in Table 1. Four studies were designed as birth cohorts [43,47-49], two were conducted in children or adolescents (age range 3-18 and 14-19 years, respectively) [46,50]. All other studies measured childhood psychosocial factors retrospectively mainly in middle-aged participants.

#### Psychosocial factors

Most of the articles provide data of parents' occupation as basic indicator variable for socioeconomic status (Table 1). Parental education was offered as main SES risk factor in three other studies. However, both SES variables were heterogeneously defined in these studies. Parents' occupation, for instance, was measured in five different classification scales ranging from two to six levels and following three different classification standards [51-53]. Childhood adversity as risk factor was found in one study only [48]. In this study, little parental interest in education was assessed as one aspect of emotional neglect. As this item might be related to formal parental education as well, results are presented in Table 1.

#### Outcomes

Self-reported diabetes was the most common outcome measure without restriction to type 2 diabetes. Only one article by Lidfeldt et al. was completely concordant with our review question by defining type 2 diabetes incidence on the basis of fasting plasma glucose levels according to ADA recommendations in an originally diabetes-free cohort [41]. HbA1c levels above 5.8% and 6.0% were used in two studies to define metabolically abnormal cases [47,48]. Insulin resistance according to HOMA-IR levels above 5.8 and 6.0 was the outcome parameter in two more studies [46,50]. One study defined "metabolically normal" cases following the definition of metabolic syndrome from the International Diabetes Federation, but without weight indicators because only participants with BMI ≥ 25 kg/m^2 ^were included [49].

### Description of included studies: Overweight and obesity

#### Study design

Thirteen studies met the inclusion criteria for obesity as outcome parameter adding up to a sample size of 70,420 participants with mainly young age groups ranging from 0-19 years. One study included middle-aged persons from 40-60 years and retrospectively assessed paternal occupation [54]. Overall, the study durations ranged from 4-33 years (median: 13 years), five studies were designed as birth cohorts [55-59].

#### Psychological factors

The risk factor definition among the obesity studies was similarly heterogeneous as in the diabetes studies. Data on parental occupation was most frequently presented followed by parental education. Family income was additionally included to parental education in four studies [58,60-62]. In one study results on the Home Observation for Measurement of the Environment Short Form Inventory (HOME-SF), a questionnaire measuring emotional and cognitive family environment and parenting abilities, was presented [61,63,64].

#### Outcomes

In most studies the outcome regarding obesity and overweight was defined according to standard cut-off values based on BMI values calculated from measured height and weight [54,56,58,60,61,65,66,68,69]. Three studies deduced BMI from self-reported anthropometric measures [55,62,67]. One study analysed dual-energy X-ray absorptiometry (DXA)-assessed fat mass [59]. Obesity incidence was determined only in the National Longitudinal Survey of Youth [61]. One study analysed age at onset of obesity and in a later publication also weight change, but without exclusion of cases with obesity at baseline [62,68]. Analysis of weight change over time was also done in three other studies [58,65,67].

### Diabetes incidence: Effect of childhood socioeconomic factors

In four studies, the risk of developing type 2 diabetes or metabolic disturbances in the offspring of lower social classes compared to children of higher status was only slightly elevated after adjustment with different effect measures (OR, HR and RR) ranging between 1.08 and 1.7 [41,42,46,48]. In the Alameda County Study, disparities in self-reported diabetes were most striking in overweight women with low childhood socioeconomic position [42]: The odds of developing type 2 diabetes was 3.2 fold higher for them than for their high SEP counterparts (OR: 2.9 (95% CI 1.7; 4.8) vs. 0.9 (95% CI 0.4; 2.2)).

As shown in Table 1, the study with the smallest population size (N = 233) showed the largest protective effect (men OR 0.2 (95% CI 0.05, 0.8); women 0.6 (0.1, 2.7)) of a high social status (father's occupation) [49]. This study was based on middle-aged participants of the Newcastle Thousand Families Cohort with a BMI exceeding 25 kg/m². Furthermore a large protective effect of maternal education (beyond elementary level) was found in a Mexican population (OR 0.6 (95% CI 0.5; 0,8)) [44]. In contrary, low maternal education at high school level or less was a significant factor influencing type 2 diabetes incidence in the Princeton School District Study (ß-coefficient = 4.47 (SE 0.78)).

On the other hand, two studies indicated small, non-significant protective effects of low social class. In a Finnish cohort children at the age of 7 years from blue-collar families had a reduced risk (OR 0.8 (95% CI 0.48; 1.45) of being diagnosed with type 2 diabetes in comparison with their white-collar counterparts [43]. In a British Cohort only women of the lowest classes were less likely to have HbA1c levels exceeding 5.8% (OR 0.8 (95% CI 0.5; 1.4)). In men, no effect of social class was seen in this study (OR 1.1 (95% CI 1.0; 1.8)) [47].

**Figure 2:**
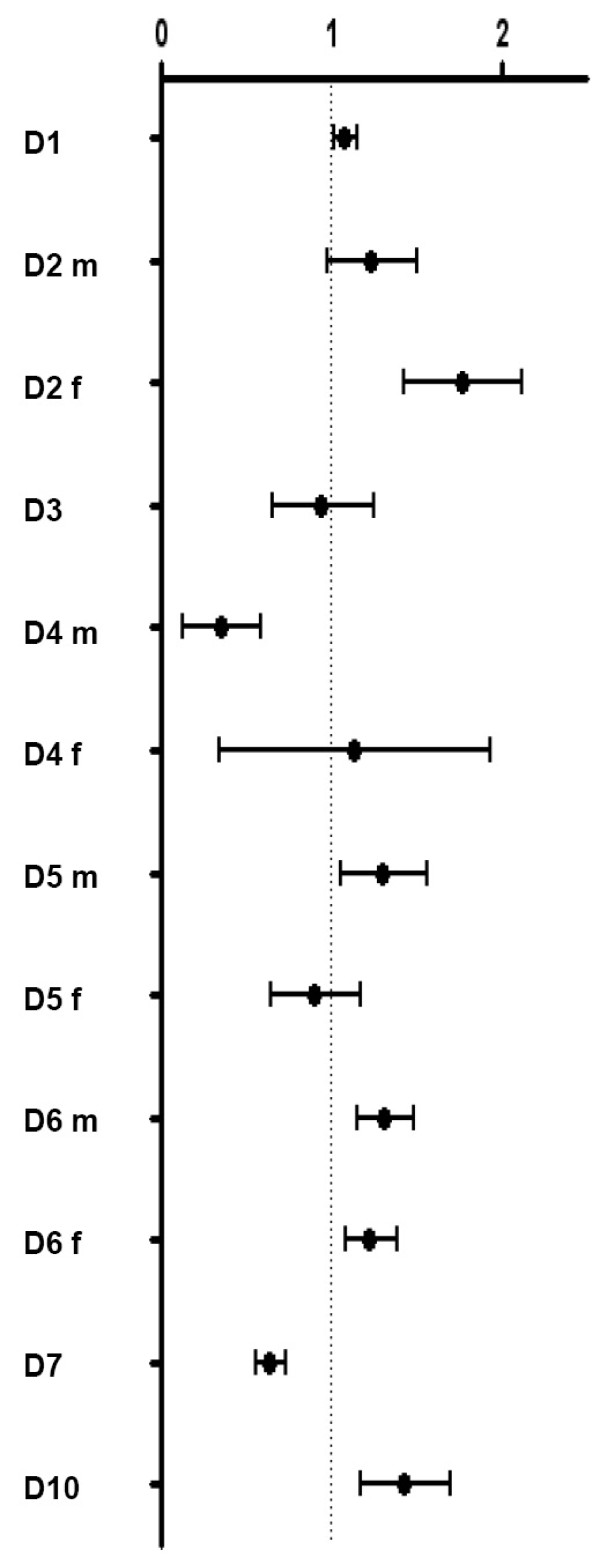
**Impact of low SES influencing the incidence of type 2 diabetes**. Effect sizes given as OR, HR or RR (central point) flanked by lower and upper 95% CI (results of high SES have been inverted, x-axis ends with 8, end points of higher results are not shown). Results of D8 and D9 are given as β-coefficients and are not included in Figure 2. D8 shows no effect of low SES; in D9 a considerably higher risk for type 2 diabetes incidence in the low SES group can be concluded.

The effect sizes observed in the included studies (except for beta coefficients) are shown in Figure 2. Effects of high social status have been inverted for better visual comparability.

### Diabetes incidence: Change in socioeconomic factors from childhood to adulthood

Furthermore, we were interested in the interaction of childhood and adult psychosocial variables. SES change was explicitly examined in the Nurses Health Study [41]. Participants in this study were relatively homogeneous with respect to their educational level and occupational status as all 100,330 participants were female nurses. Disparities emerged from different childhood socioeconomic positions and from husband's educational level. Improving SES over lifetime (spouse's high educational level and low father´s occupational status) with stable high SES as reference resulted in a slightly reduced, albeit non-significant relative risk for type 2 diabetes (RR 0.9 (95% CI 0.7; 1.3), whereas a stable intermediate SES and a declining SES influenced the type 2 diabetes relative risk negatively (RR 1.2 (95% CI 1.06; 1.4) and RR 1.18 (95% CI 1.06; 1.3), respectively). The relative risk in participants with stable low SES was comparable to those participants with stable high SES.

Langenberg et al. examined lifetime effects by analysing the change in the influence of the socioeconomic position on HbA1c levels with adjustment for childhood and adult SEP [47]. In both men and women, adult social class had stronger effects than childhood social class on HbA1c values exceeding 5.8%. In women childhood effects on HbA1c even reversed in the fully adjusted model. Langenberg et al. interpret these findings by postulating that low childhood social class continues to influence adult social class as both variables were highly correlated [47].

A high continuity of childhood and adult SEP parameters was also observed in a Finnish cohort, where children from manual classes were more likely to work in manual or lower non-manual occupations in adulthood [46].

### Diabetes incidence: Effect of other psychosocial factors

Among the retrieved studies there were several unique findings that were not comparable with other studies and other results. However, most of these studies revealed a possible association with future diabetes.

For example psychological factors such as childhood adversities [48] were associated with elevated HbA1c and obesity in one study. After full adjustment, these associations were no longer significant for elevated HbA1c. A further possible psychological risk factors for diabetes among our findings were depression and anxiety [70,71], hostility [72], and sense of coherence [73].

Also, indirect measures of socioeconomic status such as deprived neighbourhoods, and housing conditions [27,28,74,75] are likely to be relevant for future diabetes incidence. Neighbourhood characteristics also have influence on lifestyle habits depending e.g. on the availability of healthy foods and on the number of facilities for physical activity [76].

Overall, more comparable studies are needed to quantify the association of other childhood factors on metabolic impairment.

### Overweight and obesity: Effect of childhood socioeconomic factors

**Figure 3:**
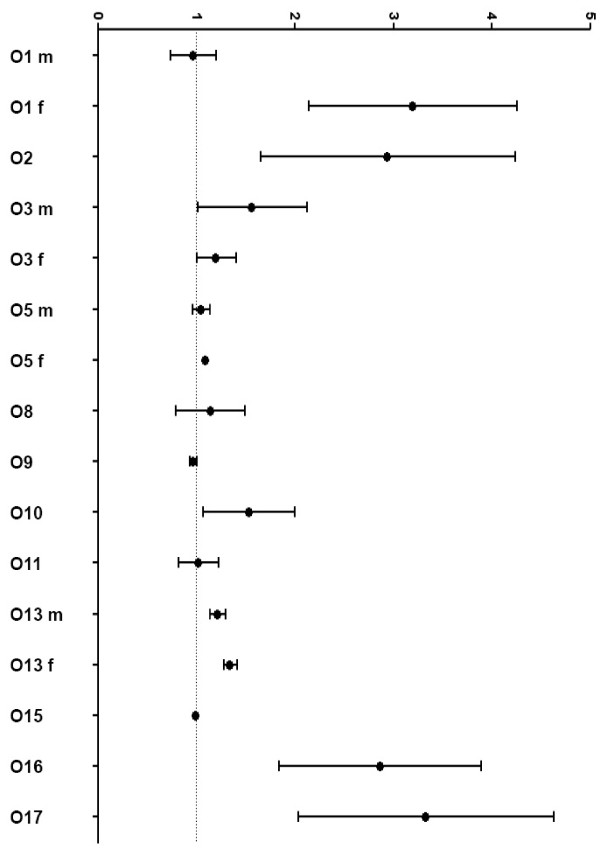
**Impact of low SES influencing overweight and obesity**. Effect sizes given as OR, HR or RR (central point) flanked by lower and upper 95% CI. Results of O6, O7, and O12 are given as β-coefficients and are not included in Figure 3. In O6 a limited effect of low SES is seen; in O7 and O12 a considerably higher risk for type 2 diabetes incidence in the low SES group can be concluded.

Table 2 gives an overview of the included studies and the observed effect sizes which are furthermore visualised in Figure 3.

Effects were most prominent in two studies regarding income discrepancies: In a national longitudinal survey in the USA the RR in the lowest intracohortal ( < 15^th ^percentile) income group for obesity incidence was 2.84 (95% CI 1.39; 5.78) compared to the highest ( > 85^th ^percentile) income group during 6 years after the baseline examination [61]. Similar results were observed in a Canadian birth cohort at the follow-up of 4.5 years [58]. At that time point the lowest income group (less than 20,000 CN$/year) had a 2.5 fold increased odds (95% CI 1.3, 4.8) of being overweight at the age of 4.5 years with reference to families with an annual income of 60,000 CN$ or more. These results were obtained after adjustment for gestational age and birth weight. One study observed that belonging to a low income family (less than 26,000 US$/year) was a significant predictor of being obese at an earlier age (β-coefficient: -2.6 (95% CI -3.8; -1.3)) [62]. No effect of family income was seen in another study which included data from the National Longitudinal Surveys Child-Mother files in the USA (OR 1.0 (95% CI 0.99, 1.00)) [57].

Parental education had no direct or a small influence on overweight or obesity outcomes in six studies [57,58,60,61,68,69]. In the National Longitudinal Survey of Youth [61], the Home Observation for Measurement of the Environment-Short Form (HOME-SF) accounted for a modulation of other CSES factors particularly for maternal education. The unadjusted OR for the six-year cumulative incidence of childhood obesity for maternal education lower than high school was 1.47 (95% CI 1.04; 2.1). The fully adjusted OR was lowered to 0.96 (95% CI 0.7; 1.4) when including also HOME-SF as covariable. In contrast, family income seemed to be independent of the HOME-SF in this study [61].

In one study regarding parental occupation gender disparities were prominent [67]. Giskes et al. found a significantly higher risk for baseline overweight and obesity (at 40-60 years) for female participants whose fathers had been working in blue-collar occupations (OR 3.4 (95% CI 1.9; 6.1), father's professional occupation as reference). This effect decreased slightly after adjustment for adult SES (OR 2.8 (95% CI 1.6; 5.2)). These women also gained significantly more weight between baseline and follow-up 13 years later (OR 2.0 (95% CI 1.7; 2.3)). In another study the risk for developing overweight during the 6-year follow-up period was increased for children from blue-collar families (OR 2.4 (95% CI 1.02; 5.4) in comparison with their white-collar counterparts [65]. Furthermore, in two studies the adjusted effects of low status were small [54,55].

Based on results of regression analysis, two studies determined low social status as factor influencing overweight and obesity [56,62], whereas in a third study no such effect was seen (ß-coefficient: -0.05 (SE 0.08)) [66]. In another study, DXA-assessed fat mass was analysed using a slope index of inequality (SII). The results of this method are comparable to regression coefficients and pointed towards a moderate association of maternal education on body fat [59].

However, as these studies only displayed data on regression coefficients, effect sizes cannot be compared to the other studies and are therefore not included in Figure 3.

Taken together, whenever effects were seen in any of the thirteen studies, they pointed towards a deleterious influence of low social status on future risk of overweight and obesity.

### Overweight and obesity: Effect of change in socioeconomic factors from childhood to adulthood

The role of change in psychosocial factors in relation to obesity was investigated in the 1958 British birth cohort [55]: Power et al. reported a continuous decrease of the effects of low socioeconomic status in childhood on obesity especially in women with an increase in social position in adulthood. The effect of low social class at the age of seven years in 33-year old women decreased from an unadjusted OR of 1.4 (95% CI 1.3; 1.6) to 1.3 (95% CI 1.1; 1.4) after adjustment for personal education [55].

### Overweight and Obesity: Effect of other psychosocial factors

We found one study which showed that traumata are associated with obesity [77]. Furthermore, depression and stress [78] are not only relevant for type 2 diabetes, but also for obesity. Also, indirect measures of socioeconomic status such as deprived neighbourhoods, housing conditions [79] and supply for healthy lifestyle habits [80], are likely to be relevant for obesity.

Therefore, psychological factors, and several measures of deprivation in childhood remain an interesting field for further examinations helping to understand the possible pathways leading to obesity and type 2 diabetes.

## Discussion

Based on the included studies and bearing in mind the limited comparability, we can state that psychosocial discrepancies in childhood seem to have an unfavourable impact on future type 2 diabetes incidence. Adjustment for adult SES and BMI attenuated these associations considerably [41,42,47]. This finding raises the question if a favourable life course may be beneficial for the participants' metabolic status. However, detailed life course analysis was rarely carried out among the retrieved studies. Only one study offered indications that improving SES over time seems protective against diabetes and that a decrease in SES is especially harmful [41]. But as only one study provided substantial data on SES change, no valid conclusion can be drawn from these results.

Furthermore, we found the family income [67,58,61] and the father´s occupation [[67], in women only; [65]] of relevance for overweight and obesity. Surprisingly, in contrast to our findings on diabetes incidence parental education seemed to have less impact on future obesity risk. However, lower parental education was linked to an earlier age at onset of obesity in one study [62].

### Limitations

Altogether we observe that for both type 2 diabetes and obesity longitudinal, population-based data are scarce. Especially, the life course including childhood indicators of psychosocial status and the role of risk factor clustering is under-investigated. Additionally, analysis of psychosocial factors is carried out heterogeneously and thus exposed to the critique of constructing SES risk factors arbitrarily. Although there are some national consensus statements especially regarding the assessment of the occupational position [51-53], it is difficult to compare these positions because they are attributed with varying amounts of prestige and are influenced by general shifts in composition [81].

### Implications for research

Three steps may help to gain further evidence on the topic: First, basic SES measures in youth and in adulthood should be consequently presented in publications. Second, for a better understanding of the role of psychosocial factors throughout life on the risk of type 2 diabetes, detailed analysis of a broad set of psychosocial factors, their interrelation and their impact on type 2 diabetes are needed. This requires a large-scale systematic analysis applying various psychosocial measures in youth and adulthood that have been found to be of relevance for diabetes and obesity such as depression [82-84], stress [85,86], unemployment [87], lifestyle habits [76,80] deprived neighbourhoods [27,28,74,75,79] and other factors [72,77].

Finally, such a large-scale systematic analysis would also require the application of different analytic approaches. As an example, the interpretation of the results of regression models analysing closely related risk factors throughout lifetime is controversial. Attenuation after adjustment for various risk factors may be attributable to a strong correlation of these risk factors [22] rather than to confounding. An interesting alternative approach may be the analysis of path models. For example Lehman et al. showed that childhood SES had a direct impact on metabolic functioning in the participants by applying path models on data from the Coronary Artery Risk Development in Young Adults study (CARDIA). Furthermore, childhood SES had an impact on early family environment, psychosocial functioning and adult SES. Interestingly, adult SES in this study had no direct influence on metabolic functioning [88]. Additionally, cluster and discriminance analysis can shed further light on the interaction of a broad set of childhood and adult psychosocial indicators accounting also for highly differentiated social milieus with distinct beliefs, behaviours and tastes [89].

### Implications for practice

Valid results on the association of childhood socio-economic circumstances and future risk of diabetes and obesity would be important to design targeted and more efficient prevention strategies. Diabetes and obesity prevention may not only profit from educational programmes but also from health politics, from interventions for high-risk families, from coping skills training [90], from empowerment of social networks and from healthy neighbourhoods [91,92].

## Conclusion

Taken together, despite the lack of homogeneous data, there is evidence for adverse effects of low psychosocial position in childhood on the risk for type 2 diabetes and obesity in later life. However, more studies and homogeneous standards regarding assessment of exposure and outcome variables and statistical analyses are needed.

## Competing interests

The authors declare that they have no competing interests.

## Authors' contributions

WR and TT conceived the study aims and design. TT, CH and WR contributed to the systematic review, data extraction, quality assessment and interpretation of the results. TT wrote the manuscript, supervised by CH and WR. All authors approved the final version of the manuscript.

## Pre-publication history

The pre-publication history for this paper can be accessed here:

http://www.biomedcentral.com/1471-2458/10/525/prepub
